# Hesitancy towards the COVID-19 vaccine among health care practitioners in the Kingdom of Saudi Arabia: a cross-sectional study

**DOI:** 10.12688/f1000research.74575.4

**Published:** 2023-03-27

**Authors:** Abdullah A. Almojaibel, Khalid Ansari, Yahya A. Alzahrani, Saleh A. Alessy, Faraz A. Farooqi, Yousef D. Alqurashi

**Affiliations:** 1 Respiratory Care Department, Imam Abdulrahman Bin Faisal University, Dammam, Saudi Arabia; 2 Department of Public Health, Saudi Electronic University, Riyadh, Saudi Arabia; 3 College of Dentistry, Imam Abdulrahman Bin Faisal University, Dammam, Saudi Arabia

**Keywords:** vaccine acceptance, COVID-19 vaccine, coronavirus, Saudi Arabia, vaccine hesitancy

## Abstract

Background: The coronavirus disease 2019 (COVID-19) pandemic is a major public health crisis worldwide. In less than 12 months since the World Health Organization declared the outbreak, several different COVID-19 vaccines have been approved and deployed mostly in developed countries since January 2021. However, hesitancy to accept the newly developed vaccines is a well-known public health challenge that needs to be addressed. The aim of this study was to measure willingness and hesitancy toward COVID-19 vaccines among health care practitioners' (HCPs) in Saudi Arabia.

Methods: A cross-sectional study using an online self-reported survey was conducted among HCPs in Saudi Arabia between April 4th to April 25th 2021 using snowball sampling. Multivariate logistic regression was employed to identify the possible factors affecting HCPs’ willingness and hesitancy to receive COVID-19 vaccines.

Results: Out of 776 participants who started the survey, 505 (65%) completed it and were included in the results. Among all HCPs, 47 (9.3%) either said “no” to receive the vaccine [20 (4%)] or were hesitant to receive it [27 (5.3%)]. Of the total number of the HCPs, 376 (74.5%) already received the COVID-19 vaccine, and 48 (9.50%) were registered to receive it. The main reason of agreement to receive the COVID-19 vaccine was “wanting to protect self and others from getting the infection” (24%).

Conclusion: Our findings have shown that hesitancy toward receiving COVID-19 vaccines among HCPs in Saudi Arabia is limited and therefore may not be a serious issue. The outcomes of this study may help to understand factors that lead to vaccine hesitancy in Saudi Arabia and help public health authorities to design targeted health education interventions aiming to increase uptake of these vaccines.

## Introduction

The coronavirus disease 2019 (COVID-19) pandemic is a major public health issue worldwide. Up to November of 2022, 628 million confirmed cases and over six million deaths were registered worldwide. ^1^ This led to development of vaccines in an expected way. For example, in less than 12 months since the World Health Organization (WHO) declared the outbreak, several numbers of COVID-19 vaccines have already been approved and deployed mostly in developed countries since January 2021. In the Kingdom of Saudi Arabia (KSA), four vaccines have been approved by the health regulatory bodies (i.e., Oxford-AstraZeneca, Johnson & Johnson’s Janssen, Moderna, and Pfizer/BioNTech), ^2^ with a priority to vaccinate health care practitioners (HCPs) alongside other groups who are at a higher risk of COVID-19. ^3^

HCPs are more likely to be infected by COVID-19 virus as they are in direct contact with infected patients. In a study by Nguyen *et al.* (2020) conducted on more than 200,000 HCPs, they found that HCPs at the frontline of care had a threefold increased risk of being infected with COVID-19. ^4^ Asua and colleagues (2021) reported that COVID-19 infection rates among HCPs have ranged between 10% and 20% in Spain. ^5^ In KSA, Barry *et al.* (2021) concluded that almost 13% of the HCPs have been infected with COVID-19. ^6^ Because of the difficulty in applying social distancing in health care facilities, HCPs might spread the virus within themselves and in between patients. This situation could be worsened in case of the shortages of personal protection equipment. ^7^^,^^8^

Multiple countries have decided to initially provide COVID-19 vaccines to the most vulnerable groups including HCPs. ^5^^,^^6^^,^^9^^–^^11^ Achieving a high vaccination coverage level among HCPs will ensure the presence of an adequate number of protected workforces to deal with the pandemic more effectively and efficiently. ^12^ Prioritizing HCPs to receive COVID-19 vaccines is essential to keep the health care system running, protect very sick individuals during the pandemic, and to ensure the provision of the vaccines to be running for the public. ^10^

Hesitancy to accept the newly developed vaccines is a well-known public health challenge, ^13^ which might be exaggerated after documenting rare thromboembolic events among vaccinated individuals. ^14^

Also, Shekhar *et al.* (2021) reported that concerns about vaccine efficacy, adverse effects, and rapidity of the production were the most important factors affecting hesitancy to receive the COVID-19 vaccine. ^15^ Hesitancy to accept COVID-19 vaccines could also be complicated by misinformation, conspiracy beliefs, and theories that the virus is man-made and used for population control. ^16^^,^^17^

Several studies have sought to determine the level of willingness to receive COVID-19 vaccines as well as the factors influencing vaccine acceptance. COVID-19 vaccine acceptance levels were varied between studies conducted in different countries. Levels of vaccine acceptance in these studies were reported to be 63% in Hong Kong as of April 2020, ^18^ 77% in France as of July 2020, ^19^ 72% in France and French-speaking Belgium and Quebec as of November 2020, ^20^ 81% in Canada as of December 2020. ^21^ To examine this further, a study conducted in KSA prior to the development of the vaccines demonstrated that only 50% of the HCPs were willing to receive the vaccine. ^22^ Moreover, the acceptance level to receive COVID-19 vaccines among HCPs in KSA (as of November 2020) was reported to be 70%. ^6^

There has been no research conducted after the approval of the COVID-19 vaccines in KSA. Therefore, this study aimed to measure hesitancy and willingness toward COVID-19 vaccines among HCPs in Saudi Arabia.

## Methods

### Design

We conducted a cross-sectional study to assess willingness and hesitancy toward COVID-19 vaccines among HCPs in KSA. We created an online self-reported survey using the Question Pro survey tool hosted at Imam Abdulrahman Bin Faisal University (IAU). The survey was offered only in English because most of the HCPs in Saudi speak and understand English. The questions asked in the survey are available as part of the *underlying data*. ^37^ Responses were collected anonymously and no personally identifying information was collected. This study was approved by the IAU’s Institutional Review Board (IRB-2021-03-149).

### Sampling

We started the data collection via convenience sampling to HCPs and via hospital’s email lists. Also, the survey was distributed via online links posted on social media platforms (e.g., Twitter, LinkedIn, and WhatsApp). In addition, we shared the survey link with multiple health care organizations, scientific societies, and associations to send it to their HCPs/members and encourage them to further share it to reach the target population. Data were collected from April 4 ^th^ to April 25 ^th^ 2021.

### Participants

All HCPs currently working in healthcare facilities in KSA, regardless of the level of patient contact and their clinical role, were eligible to participate in the study. Informed consent was obtained from all the participants prior to starting the survey. A participation consent statement was added on the study information page as follows: “If you are a health care practitioner in Saudi Arabia and consent to participate in this survey, please proceed to the next page to start the survey.” Only those who agreed to participate where allowed to complete the survey. Proceeding to the survey page was therefore taken as consent to participate.

### Measures

The survey collected participants’ demographics and health information and assessed HCPs’ attitude and perception of COVID-19 and COVID-19 vaccines. Furthermore, the survey assessed the HCPs’ willingness to receive COVID-19 vaccines as well as hesitancy level as measured by the vaccine hesitancy scale (VHS). The VHS includes 10 items measured on a 5-point Likert scale ranging from strongly disagree to strongly agree. The VHS is developed by the WHO Strategic Advisory Group of Experts (SAGE) to capture parental attitudes, beliefs, and behaviors surrounding vaccination. ^23^ The COVID-19 vaccines hesitancy scale, ^24^ which was adopted in this study, is a modified version of the VHS. The validity and reliability of the COVID-19 VHS was established in another study. ^24^ However, we pre tested the survey with nine HCPs currently practicing in KSA to assure the clarity of the questions and to evaluate the face validity of the scale. The internal consistency of the scale used in this study was assessed by Cronbach’s alpha (0.75).

### Statistical analysis

For descriptive analysis, frequency count and percentages were calculated and presented in a table format. A bivariate analysis to evaluate the associations between HCP’s willingness to the receive COVID-19 vaccines and their demographic characteristics, awareness, and health status was done using Chi-Square/Fisher Exact where appropriate. The differences in the VHS between participants who reported their willingness to receive the vaccine and those who had no intention to receive the vaccine were determined. Responses about willingness to receive the vaccine (item 10 of the VHS) were set as the dependent variable while all the other 9 items of the VHS were set as the independent variable. All analyses were performed using SPSS 26.0 (IBM Corporation, New York, NY, United States). The level of statistical significance was set at *p* < 0.05 for this analysis.

## Results

Out of 776 participants who started the survey, 505 (65.1%) completed it and were included in the analysis. The remaining 271 did not complete the survey fully; therefore, they were excluded. The demographical characteristics of the participants are presented in Table 1. Among 505 HCPs who completed the survey, 47 (9.3%) either said “no” to receive the vaccine [20 (4%)] or were hesitant to receive it [27 (5.3%)]. Of the total number of the HCPs, 376 (74.5%) had already received the COVID-19 vaccine, and 48 (9.5%) were registered to receive it. Out of the 34 participants (6.7%) who wanted to receive the vaccine, the majority of them [20 (59%)] preferred the Pfizer-BioNTech vaccine because they believed it had fewer side effects and was more effective than AstraZeneca vaccine.

**Table 1.** Sociodemographic characteristics of the study participants (n = 505).

Demographic variables		Number (%)
**Gender**	Male	259 (51.3)
	Female	246 (48.7)
**Nationality**	Saudi	438 (86.7)
	Non-Saudi	67 (13.3)
**Age**	18-24	152 (30.1)
	25-29	98 (19.4)
	30-34	70 (13.9)
	35-39	82 (16.2)
	40-44	45 (8.9)
	45-49	24 (4.8)
	50-54	21 (4.2)
	more than 54	13 (2.6)
**Residency province in Kingdom of Saudi Arabia**	West	44 (8.7)
	Central	102 (20.2)
	Eastern	345 (68.3)
	South	10 (2)
	Northern	4 (0.8)
**Health profession**	Physician	89 (17.6)
	Nurse	61 (12.1)
	Dentist	12 (2.4)
	Pharmacist	28 (5.5)
	Other Health Care Specialists (respiratory therapy, physiotherapy, clinical nutrition, etc.)	289 (57.2)
	Technician in allied medical sciences	26 (5.1)
**Current state of health**	Excellent	246 (48.7)
	Very good	173 (34.3)
	Good	75 (14.9)
	Fair	10 (2)
	Bad	1 (0.2)
**Having chronic diseases**	Yes	88 (17.4)
	No	417 (82.6)
**Infected with COVID-19**	Yes	86 (17)
	No	419 (83)
**Family member infected with COVID-19**	Yes	424 (84)
	No	81 (16)
**Received COVID-19 vaccine**	Yes	376 (74.5)
	I have registered	48 (9.5)
	No	81 (16)
**Would you like to receive COVID-19 vaccine?**	Yes, I would like to	34 (6.7)
	I would be hesitant	27 (5.3)
	No, I would refuse	20 (4)
**Preferable vaccine to receive**	Pfizer	20 (59)
	AstraZeneca	2 (6)
	No Preference	12 (35)

The associations between the demographic characteristics of the HCPs and their willingness to receive COVID-19 vaccines is presented in Table 2. Female HCPs were less willing to receive the vaccine (47.3%) compared to male HCPs. However, no statistically significant association was found between gender and willingness to receive the vaccine ( *p* = 0.26). Significant association was only found between having excellent or good health condition and the willingness to receive the COVID-19 vaccine ( *p* = 0.03).

**Table 2.** Associations between the sociodemographic characteristics of health care professionals and willingness to receive the COVID-19 vaccines.

Variables		Agree n = 387	Not sure n = 86	Disagree n = 32	p-values
**Age**	18-24	32.3%	25.6%	15.6%	0.30
	25-29	18.1%	24.4%	21.9%	
	30-34	13.7%	12.8%	18.8%	
	35-39	15.8%	16.3%	21.9%	
	40-44	9.3%	4.7%	15.6%	
	45-49	4.9%	4.7%	3.1%	
	50-54	4.1%	5.8%	0.0%	
	more than 54	1.8%	5.8%	3.1%	
**Gender**	Male	52.7%	43.0%	56.3%	0.26
	Female	47.3%	57.0%	43.8%	
**Nationality**	Saudi	85.8%	91.9%	84.4%	0.30
	Non-Saudi	14.2%	8.1%	15.6%	
**Health profession**	Physician	17.3%	16.3%	25.0%	0.13
	Nurse	10.3%	16.3%	21.9%	
	Dentist	2.6%	2.3%	0.0%	
	Pharmacist	4.4%	8.1%	12.5%	
	Other Health Care Specialists	60.2%	51.2%	37.5%	
	Technician in allied medical sciences	5.2%	5.8%	3.1%	
**Residency province in Kingdom of Saudi Arabia**	West	8.5%	9.3%	9.4%	0.34
	Central	18.1%	26.7%	28.1%	
	Eastern	71.1%	59.3%	59.4%	
	South	1.8%	2.3%	3.1%	
	Northern	.5%	2.3%	0.0%	
**Current state of health**	Excellent	48.8%	44.2%	59.4%	0.03
	Very good	34.6%	33.7%	31.3%	
	Good	14.2%	20.9%	6.3%	
	Fair	2.1%	1.2%	3.1%	
**Having chronic diseases**	Yes	18.3%	17.4%	6.3%	0.25
	No	81.7%	82.6%	93.8%	

Table 3 represents the bivariate analysis of hesitancy scale items for the HCPs who agreed to receive the COVID-19 vaccine. The majority of the participants who agreed to receive the vaccine were found to agree (53.7%) or strongly disagree (34.6%) that “the COVID-19 vaccine is important for my health”. Also, most of the participants were found to agree (33.1%) or strongly agree (48.6%) that “COVID-19 vaccines will be very effective in preventing COVID-19”. Only 21.2% of the HCPs doubt the safety of COVID-19 vaccines, and 28.9% were neutral about the vaccine’s safety.

**Table 3.** Bivariate analysis of hesitancy scale items for health care professionals who agreed to receive the COVID-19 vaccine.

Hesitancy scale items	Strongly agree	Agree	Neutral	Disagree	Strongly disagree	p-values
The COVID-19 vaccine is important for my health	53.7%	34.6%	10.6%	0.8%	0.3%	0.001
I am in a good health; I do not need to be vaccinated against COVID-19	5.4%	4.4%	8.3%	38.2%	43.7%	0.001
The COVID-19 pandemic has been alleviated, and there is no need to be vaccinated against COVID-19	3.4%	3.6%	6.7%	40.1%	46.3%	0.001
I think COVID-19 vaccines will be very effective in preventing COVID-19	33.1%	48.6%	14.0%	2.8%	1.6%	0.001
COVID-19 vaccines can protect people (family, friends, colleagues) around me from infection	40.6%	47.5%	8.8%	2.1%	1.0%	0.001
I doubt the safety of COVID-19 vaccines	8.3%	12.9%	28.9%	32.3%	17.6%	0.001
I am worried about the possible side effects of COVID-19 vaccines	7.5%	32.0%	31.3%	20.4%	8.8%	0.001
If the COVID-19 vaccine is recommended by the government, I believe vaccination is beneficial	41.6%	45.0%	12.1%	.8%	0.5%	0.001
The recommendation for the COVID-19 vaccine by doctors, the community and other professionals has a great influence on me	35.4%	47.0%	14.7%	2.3%	0.5%	0.001

Dependent variable: willingness to receive the vaccine (Item 10 of the VHS) “I am willing to be vaccinated”.

**Figure 1.**
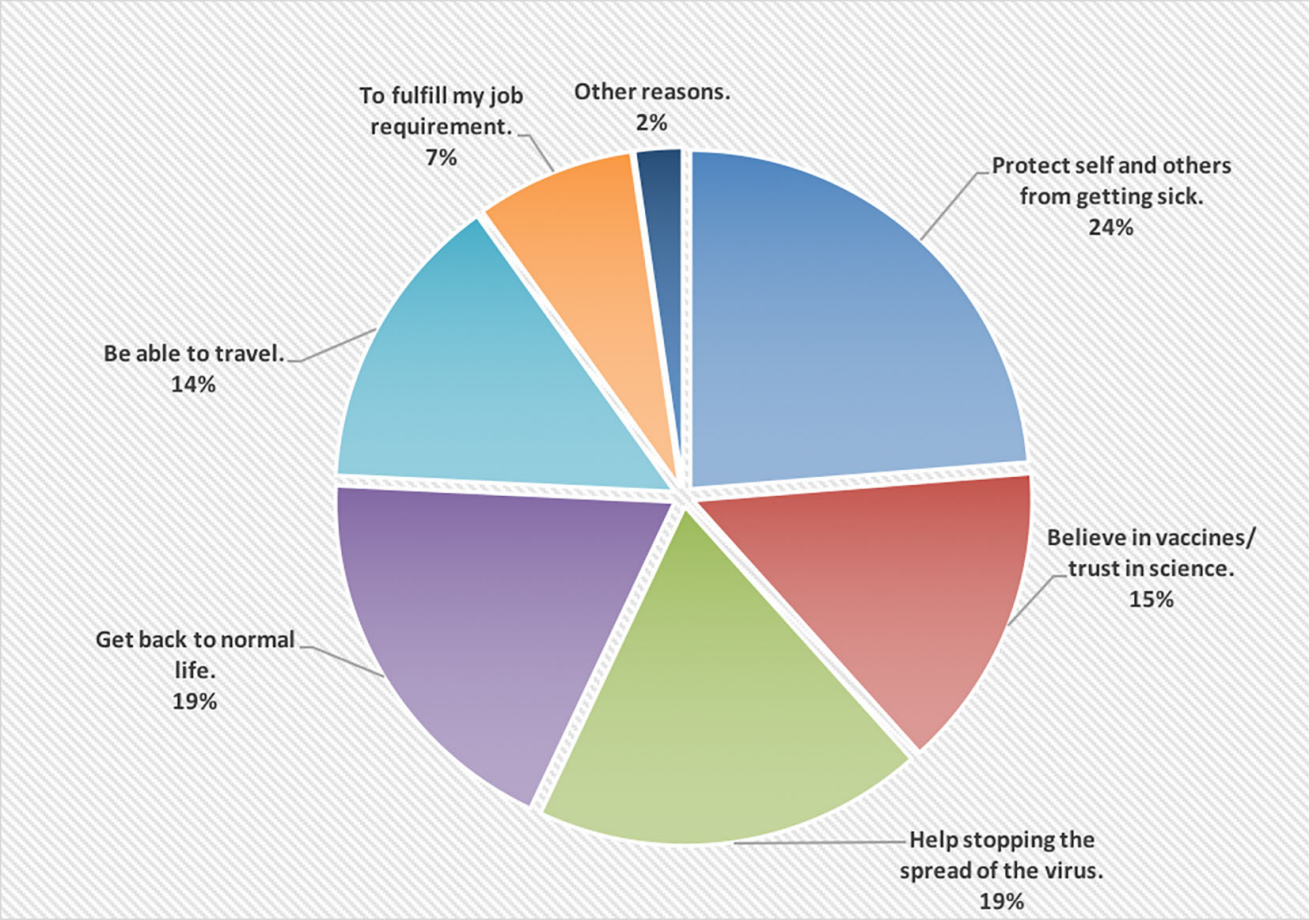
The main reasons of accepting to receive the COVID-19 vaccine.

**Figure 2.**
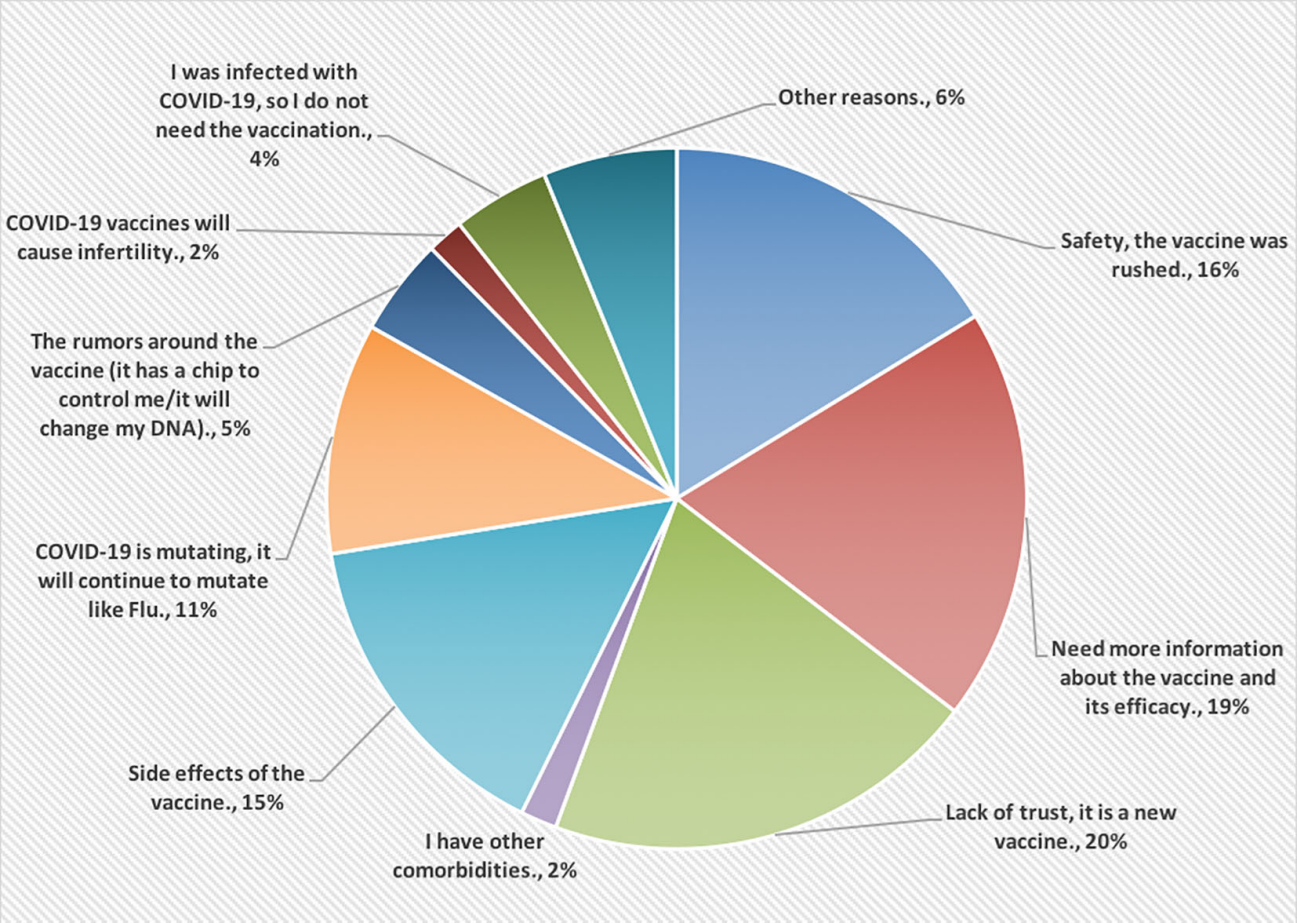
The main reasons for not accepting COVID-19 vaccines.

Figure 1 shows the reasons of accepting to receive the COVID-19 vaccine; wanting to protect self and others from getting the infection was the main reason (24%). Figure 2, however, shows the reasons for not accepting COVID-19 vaccines. Most of the HCPs were lacking the trust in this vaccine because it is new (20%).

## Discussion

The main finding of this study was that 9.3% of the HCPs either didn’t want to receive the vaccine or were hesitant to receive it. This indicates that the vaccine hesitancy among the HCPs in our sample from Saudi Arabia may not be of a serious issue. Although there are few studies assessed the hesitancy toward vaccination, our results are consistent with the current literature. ^25^^,^^26^ Civelek *et al*. (2021) found that 68.4% of physicians in Turkey were willing to get vaccinated. ^25^ Robertson *et al*. (2021) reported that 82% of UK population were willing to get vaccinated. ^26^ However, willingness level to receive the vaccine may differ between countries and communities. In a recent study, sampled from 19 countries with more than 13,000 participants, the acceptance of COVID-19 vaccines ranged from 54.8% in Russia to 88.6% in China. ^27^ Data collected from Saudi Arabia before the vaccination campaign showed that the COVID-19 vaccines’ acceptance level among the population was 64.7%. ^28^

The results of this study showed that 76.6% of the Saudi HCPs were willing to receive COVID-19 vaccines. A previous study on HCPs in Saudi Arabia, prior to the vaccination campaign, showed that the COVID-19 vaccines’ acceptance level was reported to be 50.5%. ^22^ This surge in the acceptancy level by more than 26% following the vaccine campaign can be attributed to several factors, but one major factor was that the government of Saudi Arabia prohibited unvaccinated people from traveling, entering private establishments and government buildings, or performing Hajj and Umrah. ^29^ However, our results showed that 23% of HCPs were hesitant to receive the vaccine. Not receiving the vaccine will place HCPs at higher risk of getting infected and spread the infection to their patients and other HCPs. Compliance with public safety measures (social distancing, face mask, and hygiene) could help to reduce the transmission of the virus. ^8^ However, the increased demand on health care services during the pandemic with the severe shortages in HCPs make it very challenging to comply with those measures. This is complicated by to the difficulty in implementing social distancing in health care facility and the shortages in personal protective equipment. ^8^

Our study showed that the majority of those who agreed to receive the vaccine were young, up to 24 years. This result is similar to a study conducted by Al-Mohaithef *et al*. (2020) in which they found that the majority of those who agree to receive the vaccine were from the age group between 26 to 35. ^28^ Qattan *et al*. in 2021 measured Saudi HCPs’ acceptance of the COVID-19 vaccine and found that the majority of those who agreed to receive the vaccine were from the age group between 30 to 39 years. ^22^ However, several other studies showed that the willingness to receive COVID-19 vaccines were higher in old ages (50 years and above) for HCPs, ^15^ and for the general population. ^30^ One justification for this contradiction between Saudi HCPs and others can be attributed to the youth population of Saudi Arabia compared to the western countries. In total, 37% of the Saudi population are between the age of 15 to 34 years.

Interestingly, our study results showed that the factors that influenced the HCPs willingness to receive the vaccine were:

1) Perceived their health status as excellent or very good; and
2) Believed that vaccines will relieve the pandemic.

These findings support the conclusions of several previous studies ^31^^–^^33^ that showed health issues such as mental illness, chronic health problems or physical health problems may lead to both vulnerability and inequality. ^31^ Therefore, even if the vaccines uptake falls short in some high-risk groups, a trivial increase in vaccines uptake will have significant health benefits. ^33^

We also determined the reasons for accepting or rejecting to receive COVID-19 vaccines as reported by the HCPs. Our findings contradict the results from Verger *et al*. (2021) about the safety concerns of COVID-19 vaccines. ^20^ Verger and colleagues concluded that concerns about the safety of the COVID-19 vaccines was, by far, the most important factor for hesitancy or reluctance and for moderate acceptance. ^20^ Contrarily, Shekhar *et al*. (2021) found that most HCPs (86%) believe that the COVID-19 vaccine is safe. ^15^ However, Qattan *et al*. (2021) study showed that 16.82% of the HCPs in KSA have safety and efficacy concerns about COVID-19 vaccines, and 26.73% have fear of the adverse side effects of the vaccines. ^22^ Even though our study was conducted after the beginning of the vaccine campaigns, we found that 21% of the HCPs doubt the safety of the vaccines, and 39.5% were worried about the possible side effects of COVID-19 vaccines. The increased percentage of HCPs with concerns regarding the COVID-19 vaccines in our study could be explained by the recent reports about the possible vaccine’s adverse effects, such as the formation of blood clots in large arteries. ^34^

Previous studies suggested that believing in the conspiracy theory behind COVID-19 was a factor of rejection. ^22^^,^^35^^,^^36^ This is similar to our findings which suggested that 5% of the HCPs rejected the vaccine because they believed rumors about the vaccines such as the “chip theory”. Although 5% seems low, it may reflect the fact that our population only included HCPs and this percentage could rise if we conducted the study in the general population and amongst those who do not trust any source of information on COVID-19 vaccines. However, Qattan *et al*. reported that only 0.6% of the HCPs believed that COVID-19 does not exists. ^22^

## Limitations

This study has some limitations. First, although the sample size in our study was objectively determined, we used a snowball sampling method to distribute the survey link among HCPs in the KSA. This method may have caused a selection bias since most of our sample were from the eastern province of KSA. Therefore, our sample may not be representative of all HCPs in KSA, which can limit the generalizability of the findings. In addition, this was a cross-sectional study. Therefore, we could not draw causal relationships between the factors and COVID-19 vaccine acceptance. Finally, the study's questionnaire was published online in the English language only, which produced a selection bias favoring English-literate HCPs only and those who have Internet connections.

## Future studies

Despite the limitations, our study was able to explore some of the unknown factors associated with COVID-19 vaccine acceptance and rejection which were not explored in previous studies. Also, given the representative sample size across KSA, the findings comprehensively demonstrated health care practitioners’ intention to uptake the COVID-19 vaccine. Future research is therefore needed to assess this study’s findings and to examine additional challenges around vaccinations in the Saudi population. Further investigations of the vaccine’s safety awareness and promotion strategies to encourage individuals to get the vaccine, as well as exploring key barriers towards receiving the COVID-19 vaccination are needed.

## Conclusion

Our findings have shown that hesitancy toward receiving COVID-19 vaccines among HCPs in Saudi Arabia is limited and therefore may not be of a serious issue. Also, the outcomes of this study help to understand factors that lead to vaccine hesitancy in Saudi Arabia and help public health authorities to design targeted health education interventions aiming to increase vaccine’s acceptance and uptake.

## Data availability

### Underlying data

Harvard Dataverse: Hesitancy of COVID-19 vaccine among health care practitioners in the Kingdom of Saudi Arabia, https://doi.org/10.7910/DVN/E90NQL. ^37^

The project contains the following underlying data:

- SurveyReport-8303281-04-22-2021-T042516.666.tab (raw data from questionnaire).

Data are available under the terms of the Creative Commons Zero “No rights reserved” data waiver (CC0 1.0 Public domain dedication).
