# Supplementary material for: Hesitancy towards the COVID-19 vaccine among health care practitioners in the Kingdom of Saudi Arabia: a cross-sectional study
Source: F1000Res. 2023 Jun 12;11:24. Originally published 2022 Jan 11. [Version 6] doi: 10.12688/f1000research.74575.6 (PMC10285322; doi:10.12688/f1000research.74575.6)
Supplement: Peer review history

# Peer review: Reviewer response for version 6

**DOI:** 10.5256/f1000research.151095.r177938

**Published:** 2023-06-16

**Authors:** Ali Azeez Al-Jumaili

**Affiliations:** 1 Clinical Pharmacy Department, University of Baghdad College of Pharmacy, Baghdad, Iraq

The authors addressed my comments. No more comments.

 I approve this manuscript for indexing.

Is the work clearly and accurately presented and does it cite the current literature?

Yes

If applicable, is the statistical analysis and its interpretation appropriate?

Partly

Are all the source data underlying the results available to ensure full reproducibility?

Yes

Is the study design appropriate and is the work technically sound?

Yes

Are the conclusions drawn adequately supported by the results?

Yes

Are sufficient details of methods and analysis provided to allow replication by others?

Partly

Reviewer Expertise:

Pharmacy Practice, Social and Administrative Pharmacy and Public Health

I confirm that I have read this submission and believe that I have an appropriate level of expertise to confirm that it is of an acceptable scientific standard.

# Peer review: Reviewer response for version 5

**DOI:** 10.5256/f1000research.146333.r169458

**Published:** 2023-05-05

**Authors:** Ali Azeez Al-Jumaili

**Affiliations:** 1 Clinical Pharmacy Department, University of Baghdad College of Pharmacy, Baghdad, Iraq

Dear authors,

 I disagree with your statistical analysis choice for table 3 since Chi-square cannot be done for 2*5 tables with such small numbers within the cells. I also disagree with considering Likert scale as categorical variable. It is better to redo the analysis using T-Test or binary Logistic regression. 

 Best

 Dr Ali Al-Jumaili, PhD

Is the work clearly and accurately presented and does it cite the current literature?

Yes

If applicable, is the statistical analysis and its interpretation appropriate?

Partly

Are all the source data underlying the results available to ensure full reproducibility?

Yes

Is the study design appropriate and is the work technically sound?

Yes

Are the conclusions drawn adequately supported by the results?

Yes

Are sufficient details of methods and analysis provided to allow replication by others?

Partly

Reviewer Expertise:

Pharmacy Practice, Social and Administrative Pharmacy and Public Health

I confirm that I have read this submission and believe that I have an appropriate level of expertise to confirm that it is of an acceptable scientific standard, however I have significant reservations, as outlined above.

# Peer review: Reviewer response for version 4

**DOI:** 10.5256/f1000research.145971.r167816

**Published:** 2023-03-28

**Authors:** Ali Azeez Al-Jumaili

**Affiliations:** 1 Clinical Pharmacy Department, University of Baghdad College of Pharmacy, Baghdad, Iraq

It is unclear which analysis was conducted in Table 3? Which type of regression? Please identify the outcome variable (below the table) and the beta-coefficients in a column as all regression analysis should include beta-coefficient in addition to P-value.

 Please resend the revised manuscript with new Table 3 as recommended above.

Is the work clearly and accurately presented and does it cite the current literature?

Yes

If applicable, is the statistical analysis and its interpretation appropriate?

Partly

Are all the source data underlying the results available to ensure full reproducibility?

Yes

Is the study design appropriate and is the work technically sound?

Yes

Are the conclusions drawn adequately supported by the results?

Yes

Are sufficient details of methods and analysis provided to allow replication by others?

Partly

Reviewer Expertise:

Pharmacy Practice, Social and Administrative Pharmacy and Public Health

I confirm that I have read this submission and believe that I have an appropriate level of expertise to confirm that it is of an acceptable scientific standard, however I have significant reservations, as outlined above.

# Response

**Published:** 2023-04-26

**Authors:** Abdullah Almojaibel

**Affiliations:** Imam Abdulrhman bin Faisal University, Saudi Arabia

Dear Reviewer, Thanks you for the follow up questions.

 We have mentioned in the statistical analysis section (revised) that bivariate analysis was done between the variables. The dependent variable is the response of the participants who showed willingness to receive the COVID-19 vaccine (responded with YES for item #10 of the VHS) and the independent variables were the VHS 9 items using Chi-Square test. We did not run Regression analysis. We only did the Chi-Square for associations.

 Also, we stated the dependent variable below table 3 for clarification.

 Thank You.

# Peer review: Reviewer response for version 3

**DOI:** 10.5256/f1000research.145267.r165896

**Published:** 2023-03-10

**Authors:** Ali Azeez Al-Jumaili

**Affiliations:** 1 Clinical Pharmacy Department, University of Baghdad College of Pharmacy, Baghdad, Iraq

I have one more comment: 

- Table 3. Bivariate analysis of hesitancy scale items for health care professionals who agreed to receive the COVID-19 vaccine. Which one was the dependent (outcome) variable in the regression analysis? Please clarify below the table and in the title. 

Is the work clearly and accurately presented and does it cite the current literature?

Yes

If applicable, is the statistical analysis and its interpretation appropriate?

Partly

Are all the source data underlying the results available to ensure full reproducibility?

Yes

Is the study design appropriate and is the work technically sound?

Yes

Are the conclusions drawn adequately supported by the results?

Yes

Are sufficient details of methods and analysis provided to allow replication by others?

Partly

Reviewer Expertise:

Pharmacy Practice, Social and Administrative Pharmacy and Public Health

I confirm that I have read this submission and believe that I have an appropriate level of expertise to confirm that it is of an acceptable scientific standard, however I have significant reservations, as outlined above.

# Peer review: Reviewer response for version 2

**DOI:** 10.5256/f1000research.141163.r161441

**Published:** 2023-02-14

**Authors:** Ali Azeez Al-Jumaili

**Affiliations:** 1 Clinical Pharmacy Department, University of Baghdad College of Pharmacy, Baghdad, Iraq

**Hesitancy towards the COVID-19 vaccine among health care practitioners in the Kingdom of Saudi Arabia: a cross-sectional study**

 Thank you for inviting me to review this manuscript. I invite authors to resubmit your manuscript after addressing all comments. Please carefully consider all issues I mentioned in a file including the authors’ responses to reviewer comments, and outline every change made point by point on the manuscript using track changes. Thanks

**Methods**

 The main sampling method was convenience. I am not sure how the authors can verify the snowballing sampling?

**Statistical analysis**

 Please clarify that the multivariable regression model was logistic regression.

**Results**

 Did the authors conduct the validity or reliability tests such as Cronbach alpha? If yes, include them in a table if no add this to the limitation section.

 Table 3 does not seem like Bivariate analysis since it includes one p-value.

 Where is the table for multivariate analysis?

Is the work clearly and accurately presented and does it cite the current literature?

Yes

If applicable, is the statistical analysis and its interpretation appropriate?

Partly

Are all the source data underlying the results available to ensure full reproducibility?

Yes

Is the study design appropriate and is the work technically sound?

Yes

Are the conclusions drawn adequately supported by the results?

Yes

Are sufficient details of methods and analysis provided to allow replication by others?

Partly

Reviewer Expertise:

Pharmacy Practice, Social and Administrative Pharmacy and Public Health

I confirm that I have read this submission and believe that I have an appropriate level of expertise to confirm that it is of an acceptable scientific standard, however I have significant reservations, as outlined above.

# Peer review: Reviewer response for version 2

**DOI:** 10.5256/f1000research.141163.r156821

**Published:** 2022-12-05

**Authors:** Mohammad Al-bsheish

**Affiliations:** 1 Healthcare Administration Department, Batterjee Medical College, Jeddah, Saudi Arabia

Dear Authors,

 Thank you for improving the manuscript.

 You did all comments, and I don't have any further comments.

Is the work clearly and accurately presented and does it cite the current literature?

Yes

If applicable, is the statistical analysis and its interpretation appropriate?

Yes

Are all the source data underlying the results available to ensure full reproducibility?

Yes

Is the study design appropriate and is the work technically sound?

Yes

Are the conclusions drawn adequately supported by the results?

Yes

Are sufficient details of methods and analysis provided to allow replication by others?

Yes

Reviewer Expertise:

Healthcare Administration, Occupational Safety and Quality Management, Nursing Science

I confirm that I have read this submission and believe that I have an appropriate level of expertise to confirm that it is of an acceptable scientific standard.

# Response

**Published:** 2023-04-26

**Authors:** Abdullah Almojaibel

**Affiliations:** Imam Abdulrhman bin Faisal University, Saudi Arabia

Thanks Dr. Mohammad Al-bsheish. 

 The research team would like to thank you for your valuable feedback and comments that improved the manuscript.

 Regards,

# Peer review: Reviewer response for version 1

**DOI:** 10.5256/f1000research.78348.r148319

**Published:** 2022-09-06

**Authors:** Sun Caijun

**Affiliations:** 1 School of Public Health (Shenzhen), Shenzhen Campus of Sun Yat-sen University, Shenzhen, China

This manuscript mainly investigated the COVID-19 vaccine acceptance and intentions among healthcare workers in Saudi Arabia. There have been many similar investigations, so the authors should compare their work with the published data and provide explanation of possible discrepancy.

 Only 505 valid participants were investigated in this survey. Please state how to calculate the minimum sample size of participants in this survey.

 Please also state the inclusion and exclusion criteria for these participants in this survey. Selection bias may exist if the participants with small sample size were recruited without reasonable inclusion and exclusion criteria.

 The study was conducted in April, 2021, but the pandemic situation and vaccination policies changed greatly during this year. This change always influenced people’s attitudes towards COVID-19 vaccination, and therefore the conclusion might be changed.

Is the work clearly and accurately presented and does it cite the current literature?

Partly

If applicable, is the statistical analysis and its interpretation appropriate?

Partly

Are all the source data underlying the results available to ensure full reproducibility?

Partly

Is the study design appropriate and is the work technically sound?

Partly

Are the conclusions drawn adequately supported by the results?

Partly

Are sufficient details of methods and analysis provided to allow replication by others?

Partly

Reviewer Expertise:

Vaccine; infectious diseases; antiviral drugs

I confirm that I have read this submission and believe that I have an appropriate level of expertise to state that I do not consider it to be of an acceptable scientific standard, for reasons outlined above.

# Response

**Published:** 2023-04-26

**Authors:** Abdullah Almojaibel

**Affiliations:** Imam Abdulrhman bin Faisal University, Saudi Arabia

**Dear Reviewer, Thanks for the feedback.**

 1- This manuscript mainly investigated the COVID-19 vaccine acceptance and intentions among healthcare workers in Saudi Arabia. There have been many similar investigations, so the authors should compare their work with the published data and provide explanation of possible discrepancy.

**In the discussion section we compared our findings with the other studies conducted in Saudi Arabia and other countries and explained the differences.**

 2- Only 505 valid participants were investigated in this survey. Please state how to calculate the minimum sample size of participants in this survey.

**There was no calculation of the minimum sample size in this study since we used a simplified-snowball sampling technique. The invited participants were requested to share the invitation link with their WhatsApp contacts and other social media platforms. Our data collection period was predetermined by certain dates. We stopped the data collection once we reach the predetermined date and reached a sample size close to a previous study conducted in Saudi Arabia by Qattan *et al*. in 2021 where they received 673 completed responses.**

 3- Please also state the inclusion and exclusion criteria for these participants in this survey. Selection bias may exist if the participants with small sample size were recruited without reasonable inclusion and exclusion criteria.

**We have mentioned the inclusion criteria for this study in the Participants section “All HCPs currently working in healthcare facilities in KSA, regardless of the level of patient contact and their clinical role, were eligible to participate in the study.” No exclusion criteria were stated.**

 4- The study was conducted in April, 2021, but the pandemic situation and vaccination policies changed greatly during this year. This change always influenced people’s attitudes towards COVID-19 vaccination, and therefore the conclusion might be changed.

**Yes, agree. We aimed to investigate vaccine acceptance in Saudi Arabia wright after the approval of the COVID-19 vaccines. Prior studies were already conducted before this milestone which provided different prospective and conclusion. However, more studies need to be conducted after the changes in the pandemic situation and the policies which have influenced HCPs’ attitude toward the vaccines.**

**Thank You.**

# Peer review: Reviewer response for version 1

**DOI:** 10.5256/f1000research.78348.r119369

**Published:** 2022-01-24

**Authors:** Mohammad Al-bsheish

**Affiliations:** 1 Healthcare Administration Department, Batterjee Medical College, Jeddah, Saudi Arabia

Dear authors, 

 Thank you for your submission of your article entitled “Hesitancy towards the COVID-19 vaccine among health care practitioners in the Kingdom of Saudi Arabia: a cross-sectional study” for F1000Research.

 Here are some comments for improvement. I hope these comments are useful!

 Title: your study aims to measure willingness and hesitancy toward COVID-19 vaccines among health care practitioners (HCPs) in Saudi Arabia. However, "willingness" is conspicuously absent from the title; I suggest adding the willingness to the title.

**Introduction**

 Due to massive changes in the number of cases every day, mentioning the date you got the statistics increases the accuracy for the readers. 

 The problem statement in the introduction needs to be enriching more by mentioning: 

- The impact of COVID-19 on HCPs by presenting governmental statistics and previous studies  
- More international and national literature on hesitancy and willingness of receiving the vaccine  
- The motive to conduct this study in particular if we consider the high percentage vaccination rate in Saudi Arabia and how the compensation mechanism of HCPs who are declining to receive the vaccine is affected, as they are able and trained to protect patients by using the safety compliance behaviours and adhering to PPE. Please see “Al-Bsheish, M., Jarrar, M. T., & Scarbrough, A. (2021). A Public Safety Compliance Model of Safety Behaviors in the Age of the COVID-19 Pandemic. INQUIRY: The Journal of Health Care Organization, Provision, and Financing, 58, 1–6”. 

**Methods **

- In the ***P*** ***articipant's*** part, you mentioned “All adults (>18 years of age) currently working in healthcare facilities in KSA” while your investigation is among HCPs! …This may confuse the readers. 
- In the ***Measures *** part, you wrote “we piloted the survey with nine HCPs currently practicing in KSA to assure the clarity of the questions and to evaluate the face and content validity of the scale on the targeted population”, did you mean pre-test? Because you examined the face and content validity, however, the pilot study investigates the reliability of the scale by Cronbach's alpha, and the minimal size to conduct it is 30 participants. Please See “Sekaran, U., & Bougie, R. (2016). Research methods for business: A skill-building approach. John Wiley & Sons”.‏ 

**Results **

- You wrote “Women were more hesitant to receive the vaccine (47.3%) compared to men”. While Table 2 shows the willingness to receive the COVID-19 vaccines. There is a difference between willingness and hesitancy. I would change this to "Women were less willing to receive the vaccine". 

**Discussion  **

- Well written and interesting, however, it is better to focus on the conspiracy theories in your introduction to enrich your problem statement in this study.   
- It is better to add the limitations and future studies in separate parts after the discussion. 

Lastly, please revise the manuscript for flow and English language edits and update any references 

 Again, thank you for your work, and good luck.  

 MAG

Is the work clearly and accurately presented and does it cite the current literature?

Yes

If applicable, is the statistical analysis and its interpretation appropriate?

Yes

Are all the source data underlying the results available to ensure full reproducibility?

Yes

Is the study design appropriate and is the work technically sound?

Yes

Are the conclusions drawn adequately supported by the results?

Yes

Are sufficient details of methods and analysis provided to allow replication by others?

Yes

Reviewer Expertise:

Healthcare Administration, Occupational Safety and Quality Management, Nursing Science

I confirm that I have read this submission and believe that I have an appropriate level of expertise to confirm that it is of an acceptable scientific standard, however I have significant reservations, as outlined above.

## References

[1] : A Public Safety Compliance Model of Safety Behaviors in the Age of the COVID-19 Pandemic. *INQUIRY: The Journal of Health Care Organization, Provision, and Financing* .2021;58: 10.1177/00469580211031382 10.1177/00469580211031382 PMC828735234261334

[2] : Research Methods for Business: A Skill-Building Approach. *Wiley* .

# Response

**Published:** 2022-11-15

**Authors:** Abdullah Almojaibel

**Affiliations:** Imam Abdulrhman bin Faisal University, Saudi Arabia

**Dear reviewer,**

 We would like to thank you for your valuable feedback. 

 We have modified the manuscript based on your suggestions. The introduction section is now modified with more literature about COVID-19 from KSA and other countries presenting the issues, and more statistics. All other valuable comments from the reviewer were also addressed/corrected in this version. Based on the additional citations, the reference list is now modified too.  

 Regards,
